# The correct way Celiac Disease diagnosis passes through DGP IgG levels in children 

**Published:** 2022

**Authors:** Roberto Assandri

**Affiliations:** *Clinical Investigation Laboratory, Ospedale Maggiore di Crema, Crema, Italy*

 The Oslo definition criteria define Celiac Disease (CD) as a multiple, systemic immune-mediated pathology triggered by the ingestion of gluten and related proteins in genetically predisposed individuals (1).

Antibodies against tissue transglutaminase (tTG), endomysium (EmA), and deamidated gliadin peptide (DGP) in both IgA and IgG classes represent the serological hallmarks available for CD diagnosis (1, 2). Their massive application in clinical practice has changed the CD diagnostic algorithm previously focused on the small intestinal biopsy in both children and adults. 

The literature, however, shows conflicting and incomplete data regarding their sensitivity, specificity, and predictive value in a specific patient group, namely infants and children. Indeed, the screening algorithm applying to children under two years of age is considered a controversial issue, particularly regarding the use of TTG IgA and its sensitivity (2-4). The three major international diagnostic societies, the European Society for Pediatric Gastroenterology, Hepatology, and Nutrition (ESPGHAN) the North American Society for Pediatric Gastroenterology, Hepatology, and Nutrition (NASPGHAN), and the American College of Gastroenterology (ACG), recommended the use of tTG IgA as the most cost-effective and accurate screening test for diagnosing CD (1-3). However, ESPGHAN recommends initial testing with only the TTG IgA and total IgA combined tests (2), while NASPGHAN and ACG recommend the combined use of TTG IgA and DGP IgG to improve the diagnostic accuracy (3, 4).

Their disagreement is certainly evident. Fortunately, the literature provides evidence for building reasonable and subsequent proposals.

In their recent work, Assandri et al. demonstrated that DGP-IgG precedes tTG-IgA seroconversion in children under two years of age in 80% of cases (5). Furthermore, they also evidenced that the combined use of tTG-IgA and DGP-IgG can upgrade the diagnostic sensitivity from 50% to 92% (5). More importantly, the lack of tTG-IgA with the only presence of DGP-IgG is the typical serological picture in these little patients (5). The presence of DGP-IgG only raises the question of which mechanism is behind it. This situation could be explained by the physiological immaturity of immune system and the consequently predominant IgG response in children.

A very recent meta-analysis appearing in *Nutrients* supported this evidence. Catassi et al. showed that DGP-IgG had slightly higher sensitivity than tTG-IgA (6). Furthermore, some children with early CD are missed when the DGP IgG test is not used. Meta-analysis suggests that the addition of DGP IgG may increase diagnostic sensitivity, especially in patients with a strong clinical suspicion (6). 

Moreover, The ESPGHAN recommended the use of the DGP-IgG test only in a selected patient group with a low total IgA level (<20mg/dL) (6). Assandi et al. showed a study population with a mean IgA level > 20g/dL, in which only five subjects had an IgA level lower that 20 mg/dL (5).

These considerations and the evidence strongly suggest the combined use of TTG IgA and DGP-IgG in children under two years of age.

In conclusion, revising the ESPGHAN guidelines could be suggested in the effort to standardize the recommendations worldwide.

## Conflict of interests

The authors declare that they have no conflict of interest.
